# Improving Long-Read Somatic Structural Variant Calling with Pangenome and *De Novo* Personal Genome Assembly

**DOI:** 10.1158/2767-9764.CRC-25-0769

**Published:** 2026-09-29

**Authors:** Qian Qin, Jakob M. Heinz, Heng Li

**Affiliations:** 1 Department of Data Science, Dana-Farber Cancer Institute, Boston, Massachusetts.; 2 Department of Biomedical Informatics, Harvard Medical School, Boston, Massachusetts.

## Abstract

Accurate detection of mosaic and somatic structural variants (SV) provides early diagnostic and therapeutic evidence for cancers. Although long-read whole-genome sequencing leads to more accurate SV detection than short-read sequencing, existing long-read SV callers only look at alignment against a single reference genome and are susceptible to systematic false discovery caused by germline differences between the individual genome and the reference genome. In this study, we develop a new SV filtering method that jointly considers the alignment against a pangenome and the *de novo* assembly of the germline genome. It dramatically reduces false-positive mosaic and somatic SVs in cancer cell lines with little loss in sensitivity for existing long-read SV callers. Our study highlights the essential need for pangenome or personal genome assembly to integrate SV calls for both SV discoveries and clinical diagnostics.

**Significance:**

We introduced a novel long-read SV filtering method that leverages pangenome and personal genome data and greatly improves the accuracy of somatic SV calling.

## Introduction

Structural variants (SV) are a broad spectrum of genetic variants longer than 50 bp, including interchromosomal translocations. They can be classified as simple types such as deletion, insertion, inversion, duplication, and translocation or complex types involving multiple variations in one mutation event, such as chromoplexy and chromothripsis (1). Replication stress, virus integration, and cellular processes to repair double-strand DNA breaks, such as homology-directed repair, can mechanistically lead to most of the SV events (2). These SV events form major sources of genome mutation and instability, hallmarks in cancer (3, 4). A recent pan-cancer whole-genome sequencing (WGS) project has enabled a more comprehensive overview of the prevalence of somatic SVs in cancer and discovered that 55% of driver mutations represent SVs, and driver SVs are more prevalent than point driver mutations in some cancers, such as breast and ovarian adenocarcinomas (2, 5, 6).

Traditional techniques to identify SVs, such as fluorescence *in situ* hybridization, have low throughput and can only analyze a few loci. Short-read WGS has genome-wide coverage and can capture SVs across the genome. However, it has notable genome coverage bias (7, 8) and is inferior for SV calling (9). Long-read sequencing, nominated as the method of the year in 2022 (10), is promising to fill these gaps with reduced coverage bias (11) and improved mappability.

Several long-read SV callers have been optimized for somatic SV calling for tumor–normal pairs, including Severus (12), SAVANA (13), SVision (14), Sniffles2 (15), and nanomonsv (16). They take a read alignment from a tumor sample and a read alignment from a normal control as input to find SVs specific to the tumor sample. All these SV callers only consider alignments against a single reference genome. At a germline insertion/deletion (indel) relative to the reference, it is possible that a small number of reads from the tumor are misaligned due to the indel and look like a different SV, but reads from the normal control are not misaligned by chance. This would lead to a potential false somatic SV call, as somatic events are generally supported by fewer reads. Current SV callers have to introduce heuristics, such as filtering out variable number tandem repeat (VNTR) regions, to alleviate the issue, but these hacks are not solving the root problem: false somatic SV calls caused by misalignment around germline SVs.

Mosaic SV calling from normal tissues is even more challenging. It is hard to distinguish mosaic and germline SVs in the absence of a sample without mosaic events. At present, only Sniffles2 calls mosaic SVs. However, it calls hundreds of mosaic SV calls in normal samples, the great majority of which are false positives (FP). There are no capable somatic or mosaic SV callers without matched normal samples.

In this article, we will describe a new filtering method to greatly reduce false somatic or mosaic SV calls caused by germline SVs. This method jointly considers alignment against the *de novo* assembly of the normal sample or against a pangenome, in addition to the alignment against a single reference genome. Because an ideal *de novo* assembly encodes all germline SVs and a pangenome represents most common germline SVs in the human population, we can reduce misalignment caused by germline SVs and thus improve somatic or mosaic SV calling accuracy. Our filtering method can be applied jointly with major existing SV callers. Evaluated on six tumor–normal pairs of cell lines as true-positive (TP) cases, the method greatly reduces FP somatic SV calls at little cost to sensitivity.

## Materials and Methods

### Method overview

We developed a command-line tool, minisv, that filters existing somatic SVs, evaluates SV calls, and constructs the union call set from multiple callers. Minisv is unique in that it can jointly consider the read alignment against multiple genomes or pangenomes to reduce alignment artifacts caused by germline SVs not present in a single reference genome. This feature is particularly powerful when the normal sample in a matched tumor–normal pair can be *de novo* assembled. The pangenome reference used here is constructed from 464 Human Pangenome Reference Consortium's (HPRC) Release 2 (HPRC2; bioRxiv 2026.07.21.739710; ref. 17) assemblies from diverse populations.

**Figure 1.**
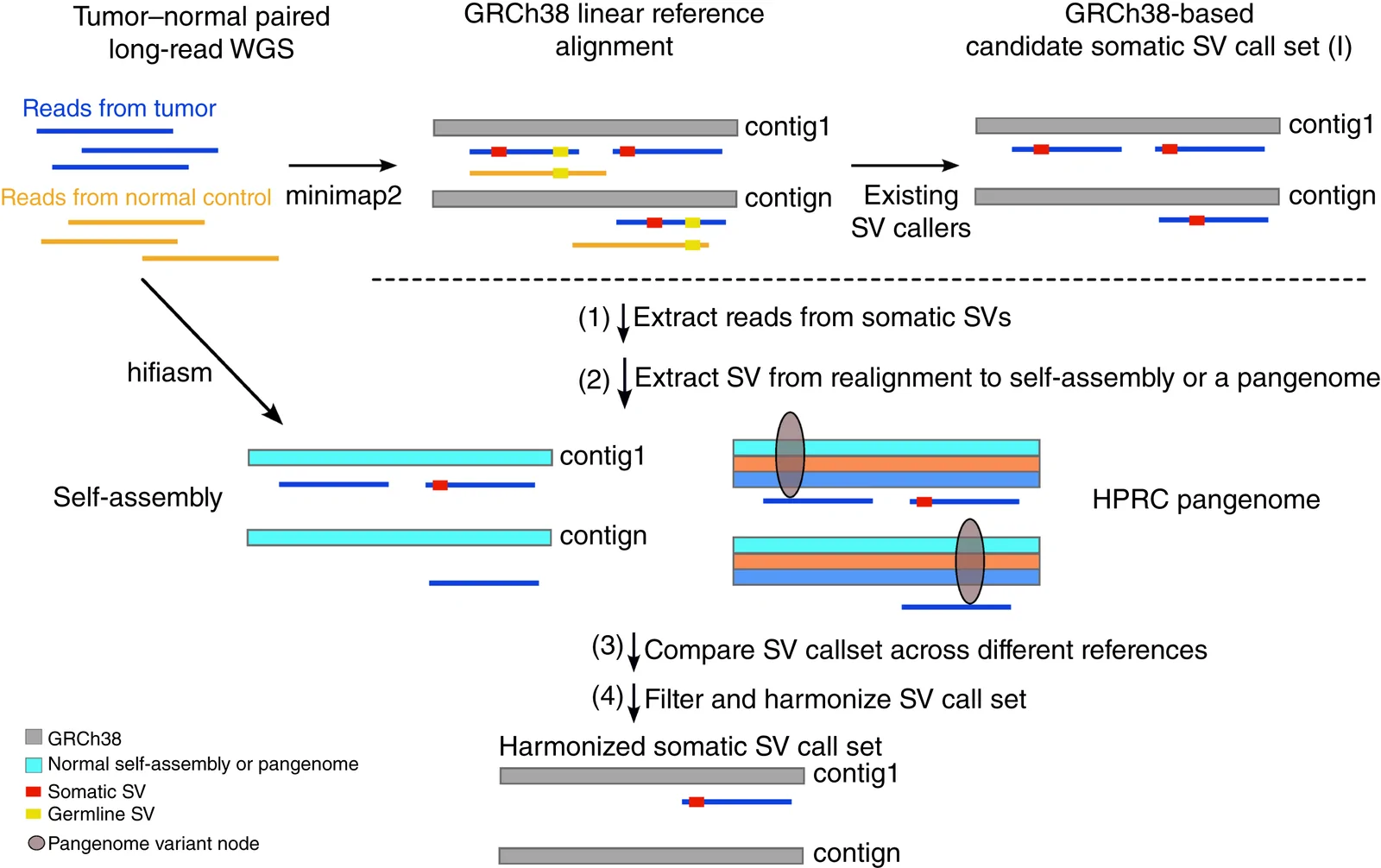
Overview of somatic SV calling and filtering workflow. Minisv starts from the existing somatic SV call set. Step (1) Extract reads from somatic SVs in the tumor from the call set; (2) realign SV-containing reads to GRCh38, normal self-assembly (if available), and/or pangenome and extract candidate SVs from the realignment in the read coordinate; (3) compare the candidate SVs resulting from the alignment against multiple genomes; and (4) for each SV call set, extract supporting reads and filter a read if its alignment to the self-assembly and/or pangenome does not contain the SV. Given multiple SV callers, minisv can generate a union somatic call set. Gray boxes denote the reference genome such as GRCh38; cyan boxes denote contigs from self-assembly; short red bars indicate potential somatic SVs; thin blue lines correspond to reads from the tumor aligned to the contigs of different reference genomes; thin orange lines correspond to reads from the normal control; and large brown circles indicate pangenome variant nodes that vary across individuals.

After getting multiple SV call sets from existing SV callers, minisv filters somatic SVs in four steps (Fig. 1): (i) for each SV call set, extract SV-containing reads with seqtk (https://github.com/lh3/seqtk); (ii) for each read, extract candidate SVs from its alignment against the linear reference genome (GRCh38), the pangenome graph, and the self-assembly of the normal sample if available; (iii) for each read, compare candidate SVs extracted from each input alignment and only retain an SV if the same SV in the read coordinate is observed across all alignments; and (iv) filter each call set based on the remaining candidate SVs across reads and harmonize SVs across call sets. This procedure leads to a nonredundant SV call set that is supported by a linear reference genome, a pangenome, and paired normal self-assembly across all callers.

### Producing self-assembly

For a pair of matched tumor and normal samples, the normal sample is *de novo* assembled with hifiasm (18) v0.19.6 using PacBio High-Fidelity (HiFi) reads. This assembly is called self-assembly. A more recent hifiasm (19) v0.24.0 is used for the assembly of Oxford Nanopore Technology (ONT) sequencing simplex reads with the option “–ont.”

### Aligning long-read sequences

HiFi reads were aligned to GRCh38 and self-assembly by minimap2 (v2.28; ref. 20) with the option “-ax map-hifi -s50” and aligned to the CHM13 pangenome graph by minigraph (v0.21; ref. 21) with the option “-cxlr.” Long-read alignments are stored in PAF and GAF formats. ONT reads were aligned to the same set of references but with a different minimap2 preset “lr:hq.”

### Extracting candidate SVs

The *extract* subcommand of minisv takes unsorted long-read alignment as input and detects candidate SVs in each read based on indels indicated by alignment CIGAR and breakpoints from split alignment records. By default, it ignores an alignment if it meets one of the following conditions: (i) low fraction of aligned bases (<0.7), short alignment length (<100 bp), low alignment length on each end (<2,000 bp), and low alignment length in the middle (<50 bp); (ii) low mapping quality (<5) and low mapping quality on each end (<30); or (iii) excessive number of ≥50 bp indels per read (≥3 per 10 kb). Indels spanning two different pangenome segments are also ignored. These thresholds aim to filter extreme cases and do not greatly affect final results.

Minisv classifies each candidate SV as a deletion, insertion, inversion, duplication, translocation, or breakend. Each candidate SV is further annotated by chromosome (or graph genome contig), start, end, strand, breakpoints offset on the reference and the read, mapping quality, distance to centromere, read name, and SV type. For extracting SV signatures from self-assemblies, we allowed the mapping quality of long-read alignment to be zero.

### Integrating candidate SVs from multiple genomes

The *isec* subcommand of minisv combines candidate SVs extracted from alignment against multiple genomes or pangenomes, which in this article include GRCh38 (labeled as “l” for linear), CHM13 graph (“g” for graph), and self-assembly (labeled as “s”). Suppose a candidate SV is observed at position *p* of a read in its alignment to GRCh38. The *isec* subcommand retains the SV on the read coordinate if the SV is observed within the 2 kb window around position *p* on the same read in its alignment against another genome or pangenome; *isec* drops the candidate SV otherwise.

### Simulating mosaic SVs

Tumor HiFi reads are downsampled with samtools and mixed with normal HiFi reads to a ratio of 1:10 on average across the genome. The tumor-to-normal ratio of a particular SV may deviate from the average due to statistical fluctuation. The simulated mix is assembled to produce the self-assembly.

### Generating somatic SV calls with existing SV callers

Existing callers were applied to the alignment against GRCh38 to call somatic SVs (Fig. 1). Severus (v1.6) requires phased alignment as input. Following its documentation, we called SNPs and short indels in normal samples with Clair3 using the option “--enable_phasing --longphase_for_phasing.” We used the pretrained model “hifi_revio” for HiFi and “r1041_e82_400bps_sup_v430” for ONT. We then applied “whatshap haplotag” to generate phased alignment. We ran Severus in the VNTR-aware mode, which generally improved SV accuracy. We used the same phased alignment as input to SAVANA (v1.3.6). Nanomonsv (v0.8.0) and Sniffles2 (v2.6.3) do not use phasing. We ran Sniffles2 under the option “–tandem-repeats” to generate two intermediate “snf” files for tumor and normal samples, respectively. We then produced a two-sample VCF with Sniffles2 and selected tumor-only SV calls with “minisv snfpair.”

### Generating mosaic SV calls with existing SV callers

Sniffles2 supports mosaic SV calling with the option “--mosaic.” Severus does not natively support mosaic SV calling. We instead ran Severus in single-sample mode with and without panel of normal (PON) filtering and only retained SV calls with a variant allele fraction below 25%. SAVANA was run in tumor-only mode, providing the built-in 1000 genome variants; we further filtered the SAVANA call set based on the HPRC-derived PON (https://zenodo.org/records/16728828/files/GRCh38-464.bb.bed.gz?download=1).

### Filtering SV calls by existing callers using self-assembly and pangenome

To improve existing SV callers, we asked other SV callers to output the names of reads supporting each SV call, aligned the reads to the self-assembly, and extracted candidate SVs with minisv. We filtered out a read (and thus reduced the supporting read count by one) if the read does not contain an SV in self-assembly alignment that does not match the original SV call.

We consider an SV called from self-assembly (called “self” in brief) matching the original SV call by an SV caller (called “original”) if one of the following conditions is met: (i) both original and self SVs involve two chromosomes or contigs (i.e., both are translocations); (ii) the original SV is longer than 100 kb, and the self SV involves two contigs, which could be caused by an assembly gap; or (iii) both original and self SVs are not translocations, and they are similar in length (differences between the two SV lengths are less than or equal to 1 kb or the SV length in one reference is larger than or equal to 60% of the SV length in the other reference).

### Evaluating SV calls

The *eval* subcommand of minisv evaluates the accuracy of an SV call set against a truth SV set. It considers two SVs to be *equivalent* if (i) the endpoints of one SV are within 500 bp windows around the endpoints of the other SV and, (ii) in the case of INDELs, the difference in length is below 1 kb or 40% of the longer SV. These rules are similar to the *merge* subcommand, but the thresholds are more relaxed because different SV callers may output SVs at slightly different locations. We manually checked alternative thresholds and found the default can reliably separate correct SV calls from wrong ones. Under this equivalence definition, a truth SV without equivalent test SVs is a false negative, whereas a test SV without an equivalent truth SV is an FP. The *eval* subcommand also optionally takes a list of confident regions, which in this article exclude centromeric regions, acrocentric short arms, and pseudoautosomal regions on chromosomes X and Y. SV calls overlapping with these regions are ignored.

### Constructing ensemble call sets

The *union* subcommand constructs an ensemble call set from multiple input SV call sets. Conceptually, each SV call is considered a node in a graph, and two SVs are connected by an edge if they are equivalent according to the rules above. The subcommand performs single-linkage clustering to group SV calls from different callers. It then checks and reports whether all SV calls in a cluster are equivalent to each other. As somatic SVs are sparse in the genome, 99% of clusters are cliques—all SVs in a cluster are equivalent to each other but are not equivalent to SVs outside the cluster. This suggests that single-linkage clustering works well in practice.

### Filtering by gnomAD SVs

We downloaded the BED file for all the aggregated SV calls in gnomAD. We define the germline SV set in this cohort after applying an allele fraction cutoff >0.1% and >1%. After extracting both breakpoints given all the SVs in gnomAD, we excluded the somatic SV calls that intersected with this gnomAD germline SV set using the same criterion as in the “Evaluating SV calls” section.

## Results

### Evaluating somatic SV calling with COLO829 tumor–normal sample pairs

To evaluate the somatic SV filtering performance of our tool, we took a previously established COLO829 call set as the ground truth (22) to test and quantify the effect of self-assembly on somatic SV calling. This call set consists of 58 SVs, including 38 indels ≥100 bp, 7 fold-back inversions, and 13 translocations.

**Figure 2.**
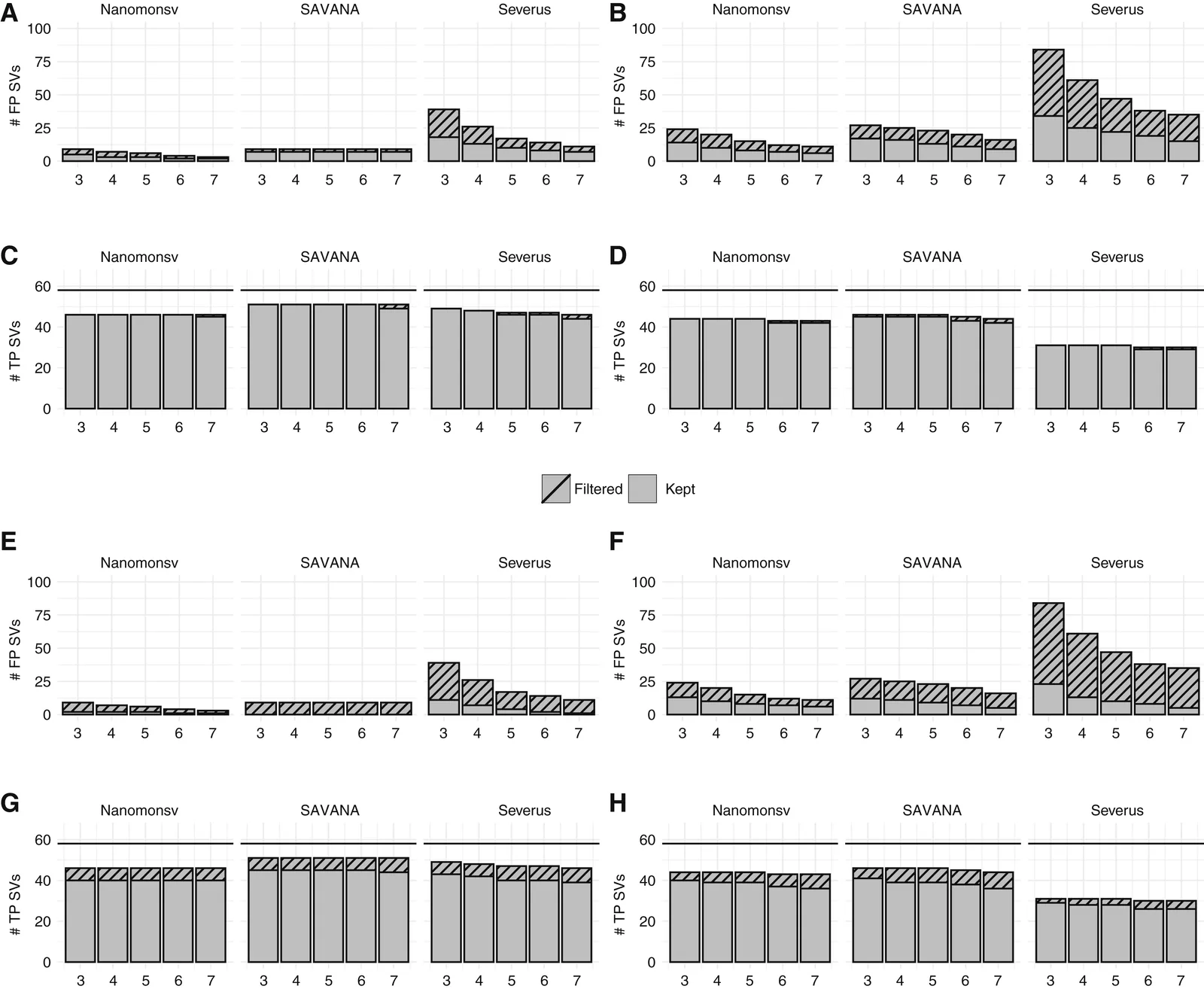
Somatic SV calling on matched COLO829 tumor–normal cell lines. **A–D,** Metrics on *de novo* assembly-based filtering. **E–H,** Metrics on pangenome-based filtering. **A** and **E,** Number of FP and TP SV calls (**C** and **G**) from PacBio HiFi data at different cutoffs on supporting reads. The solid bars correspond to SV calls still observed in the alignment against the COLO829BL (derived from blood) assembly, whereas the shaded bars correspond to calls filtered by the assembly. The horizontal line gives the number of SV calls from Valle-Inclan and colleagues (22), which are taken as ground truth. **B** and **F,** Number of FP and TP calls (**D** and **H**) from ONT reads.

We called somatic SVs from the COLO829 tumor–normal pair with Severus, SAVANA, and nanomonsv. We generated the self-assembly reference from the normal genomic reads of the paired normal sample (Supplementary Table S2). We filtered an SV call if the SV-supporting reads were aligned without SVs against the self-assembly (Fig. 2A–D). This filter barely affects TP somatic SV calls, but it greatly reduces the number of FP calls for all callers in both PacBio and ONT reads (Fig. 2A and B), and the filtering effect remains stable as we downsampled the reads from the tumor and normal control samples to half of their sequencing depths (Supplementary Fig. S1A–S1D). Among SV callers, Severus, SAVANA, and nanomonsv have broadly similar accuracy (Fig. 2). We did not include Sniffles2 in this figure because it generated many FP SVs without the self-assembly filter. Aside from this, self-assembly filtering improves the F1 score across all ranges of stratified SV size (Supplementary Tables S3 and S4).

In comparison with assembly-based filtering, pangenome-based filtering is more aggressive, removing more FPs along with TPs (Fig. 2E–H). Effectively, the pangenome-based approach filters out somatic SVs around germline SVs in the reference panel. Because germline SVs are enriched in complex regions, such as tandem repeats and segmental duplications, real somatic SVs in these regions tend to get filtered with pangenome but not with self-assembly. On the other hand, complex regions are harder to assemble and align. More aggressively filtering out these challenging regions helps reduce FPs caused by incomplete assembly or alignment artifacts. This is why pangenome-based filtering may have slightly higher specificity.

We noticed that no callers could call all SVs in the truth set and that even with the self-assembly filter, there are still a small number of SV calls not found in the truth set. We reasoned that the COLO829 cancer cell line may still be evolving across different passages of cell growth (medRxiv 2024.03.15.24304369; ref. 23) and different data centers may have generated sequencing data with different DNA materials. This may also explain the slightly higher number of extra and missing ONT calls after the self-assembly filter (Fig. 2B, D, F, and H).

We also tried gnomAD-based SV filtering and found it failed to filter out most FP SVs in COLO829 (Supplementary Fig. S2). An important caveat is that most gnomAD SVs have approximate coordinates, but designed for long reads, our method tests SV breakpoints and works best with accurate positions. Nonetheless, even if we could develop a new method specialized for gnomAD, its completeness remains a concern. Notably, HPRC2 contains 109k SVs of allele frequency >1% (allele count ≥5). A total of 33.6k are outside low-complexity regions (24). Of them, 8.1k are not within 50 bp around gnomAD SVs of >0.1% in frequency—gnomAD is missing 24% of common SVs. This would reduce the power of gnomAD for filtering germline SVs called from long reads.

### Emulating and evaluating mosaic SV calling with COLO829 data

To emulate a dataset with known mosaic SVs, we downsampled the COLO829 reads from tumor and *in silico* mixed them with the reads from normal control such that the normal-to-tumor ratio is 10:1 on average. Given approximately 60-fold tumor and normal read coverage in our data, the read coverage of the tumor sample is six across the genome. As each somatic SV only occurs in one haplotype in tumor cells, the number of reads supporting the SV is only three on average. Although some SVs may be supported by more reads due to aneuploidy and focal amplifications, we still expect SV callers to miss many somatic SVs in the *in silico* mix.

**Figure 3.**
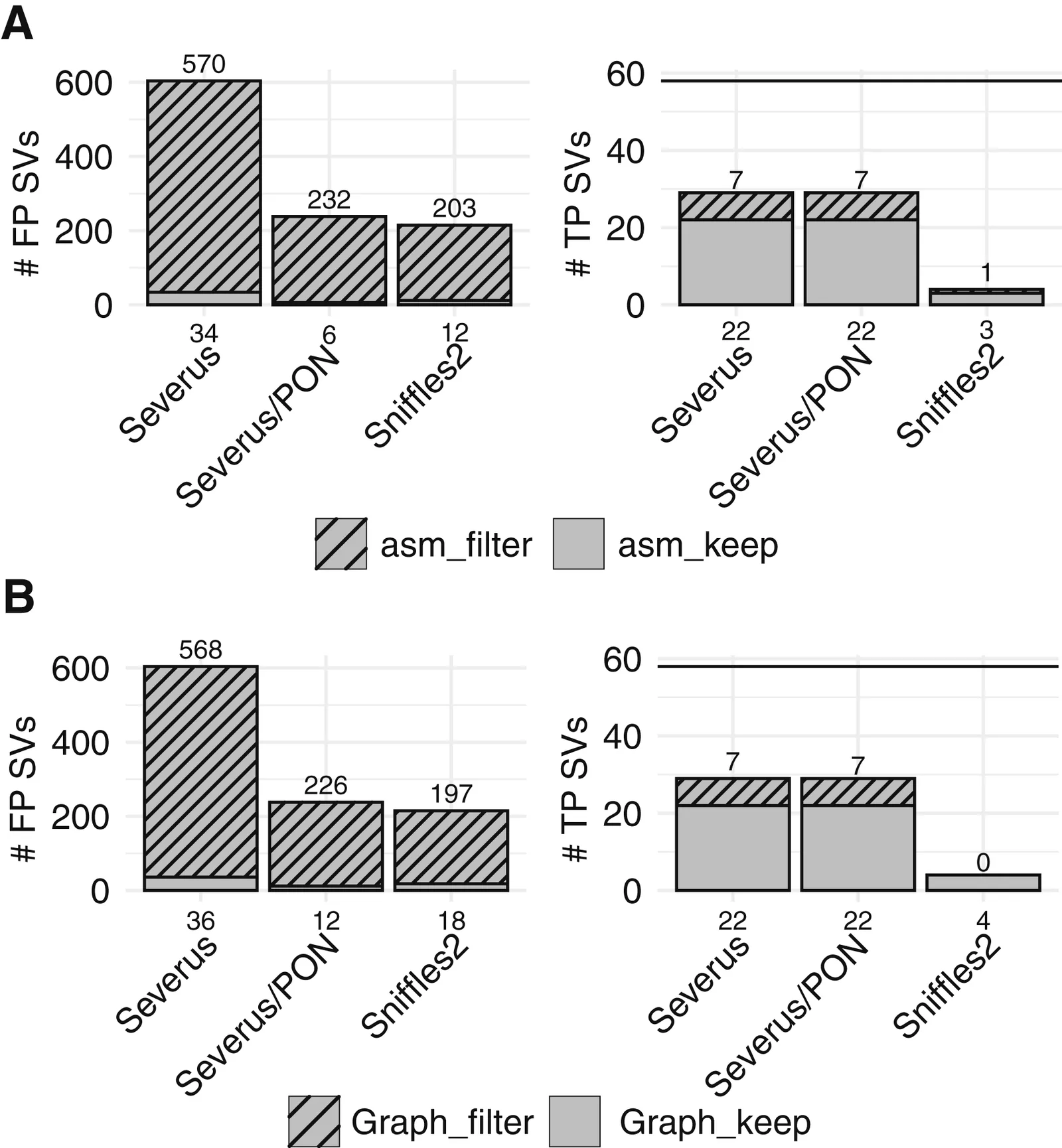
Mosaic SV calling from *in silico* mix of normal–tumor data. COLO829 tumor HiFi reads were computationally downsampled and mixed with all COLO829BL normal reads to a 10:1 normal-to-tumor ratio. **A,** The *in silico* mix was assembled and used for *de novo* assembly (asm)-based filtering. **B,** HPRC2 pangenome-based filtering.

We called mosaic SVs with three callers along with potential germline SV after filtering: (i) Sniffles2 in the mosaic mode and (ii) Severus with/without 1000G PON filtering *post hoc* allele fraction filter (“Materials and Methods”). We required each SV call to be supported by ≥2 reads. Using alignment against GRCh38 only, all three tools called hundreds of supposedly somatic SVs (Fig. 3). Most of these SVs can be found in the normal sample: they are germline SVs and are thus FP somatic calls. The self-assembly filter reduces the number of such FPs by more than an order of magnitude while retaining the great majority of TP calls. The several true somatic SVs mistakenly filtered by the self-assembly tend to reside in regions with focal amplification in COLO829. They are supported by enough reads from the tumor to be assembled into contigs. The self-assembly filter may affect sensitivity for mosaic SVs of high allele fraction.

Across different SV callers, Severus with PON and allele fraction filtering has the best sensitivity and specificity with 22 TP SVs and 6 FP SVs (Fig. 3A) with *de novo* assembly-based filtering, whereas with pangenome-based filtering, we identified the same number of TP mosaic SVs but reduced FP SVs, achieving slightly lower specificity with 12 FP SVs (Fig. 3B). With *de novo* assembly-based filtering, Sniffles2 in the mosaic mode misses most somatic SVs in the ground truth. It only detected 3 TP mosaic SVs with 12 FP SVs (Fig. 3A). We additionally tried SAVANA. It reported seven FP SVs after the self-assembly filtering, but it missed all TPs. We thus did not show the result in the figure.

### Investigating somatic SV calling for additional tumor–normal cell line pairs

**Figure 4.**
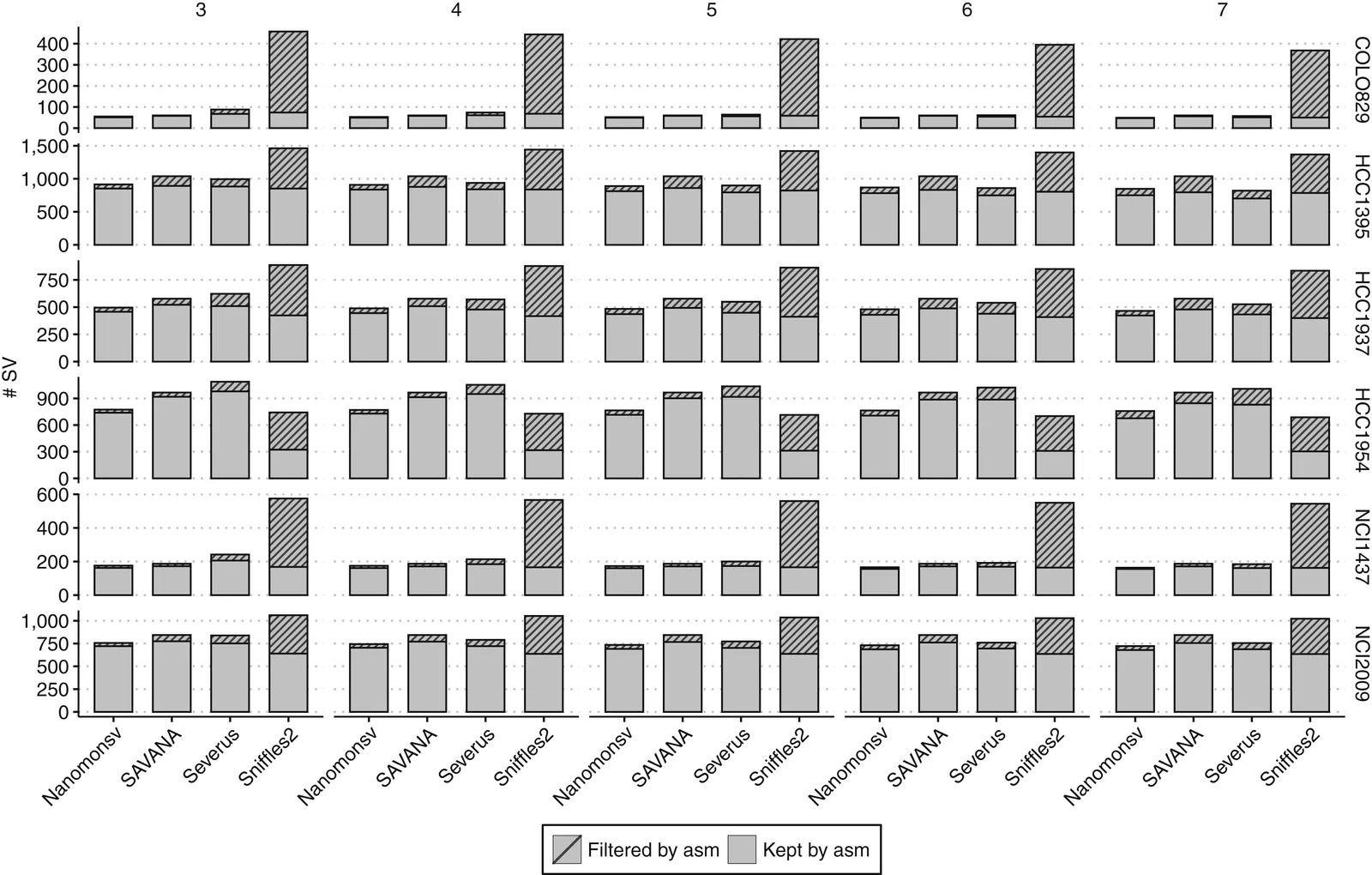
Effect of assembly (asm)-based filtering on SV calling on six tumor–normal pairs. The number on the top indicates the number of supporting reads required to call an SV.

We ran all SV callers on six tumor–normal paired cell lines, including COLO829, HCC1395, HCC1937, HCC1954, H1437, and H2009 (Table 1). As there is no curated ground truth for these cell lines, we could not compute TPs and FPs, but we could see the self-assembly filter continued to play a role in reducing somatic SV calls, especially for SVs supported by few reads (Fig. 4). These filtered SVs are likely FPs. The number of SVs called by each tool, except Sniffles2, is broadly comparable after the self-assembly filter. The effect of filtering is also apparent at halved read depth (Supplementary Fig. S3).

**Table 1.** Summary of PacBio HiFi and ONT sequencing data.

Data	Tech	RRID	Cancer sample source	Normal sample source
COLO829	HiFi	Tumor: CVCL_1137 BL: CVCL_1999	https://downloads.pacbcloud.com/public/revio/2023Q2/COLO829/	https://downloads.pacbcloud.com/public/revio/2023Q2/COLO829/COLO829-BL/
COLO829	ONT	Tumor: CVCL_1137 BL: CVCL_1999	PAU61426 at https://labs.epi2me.io/colo-2024.03/	PAU59807 at https://labs.epi2me.io/colo-2024.03/
HCC1395	HiFi	Tumor: CVCL_1249 BL: CVCL_1250	https://downloads.pacbcloud.com/public/revio/2023Q2/HCC1395/HCC1395/	https://downloads.pacbcloud.com/public/revio/2023Q2/HCC1395/HCC1395-BL/
HCC1937	HiFi	Tumor: CVCL_0290 BL: CVCL_3281	SRR28305185	SRR28305182
HCC1954	HiFi	NA	SRR28305163	SRR28305160
H1437	HiFi	NA	SRR28305179	SRR28305175
H2009	HiFi	NA	SRR28305172	SRR28305169
G100k-59	ONT	NA	EGAN50000118611	EGAN50000118609
G100k-66	ONT	NA	EGAN50000118579	EGAN50000118589
SARC-010	ONT	NA	EGAN50000118593	EGAN50000118583
SARC-016	ONT	NA	EGAN50000118575	EGAN50000118602
G100k-41	ONT	NA	EGAN50000118603	EGAN50000118580

Abbreviation: BL, B-lymphoblast cell line.

**Figure 5.**
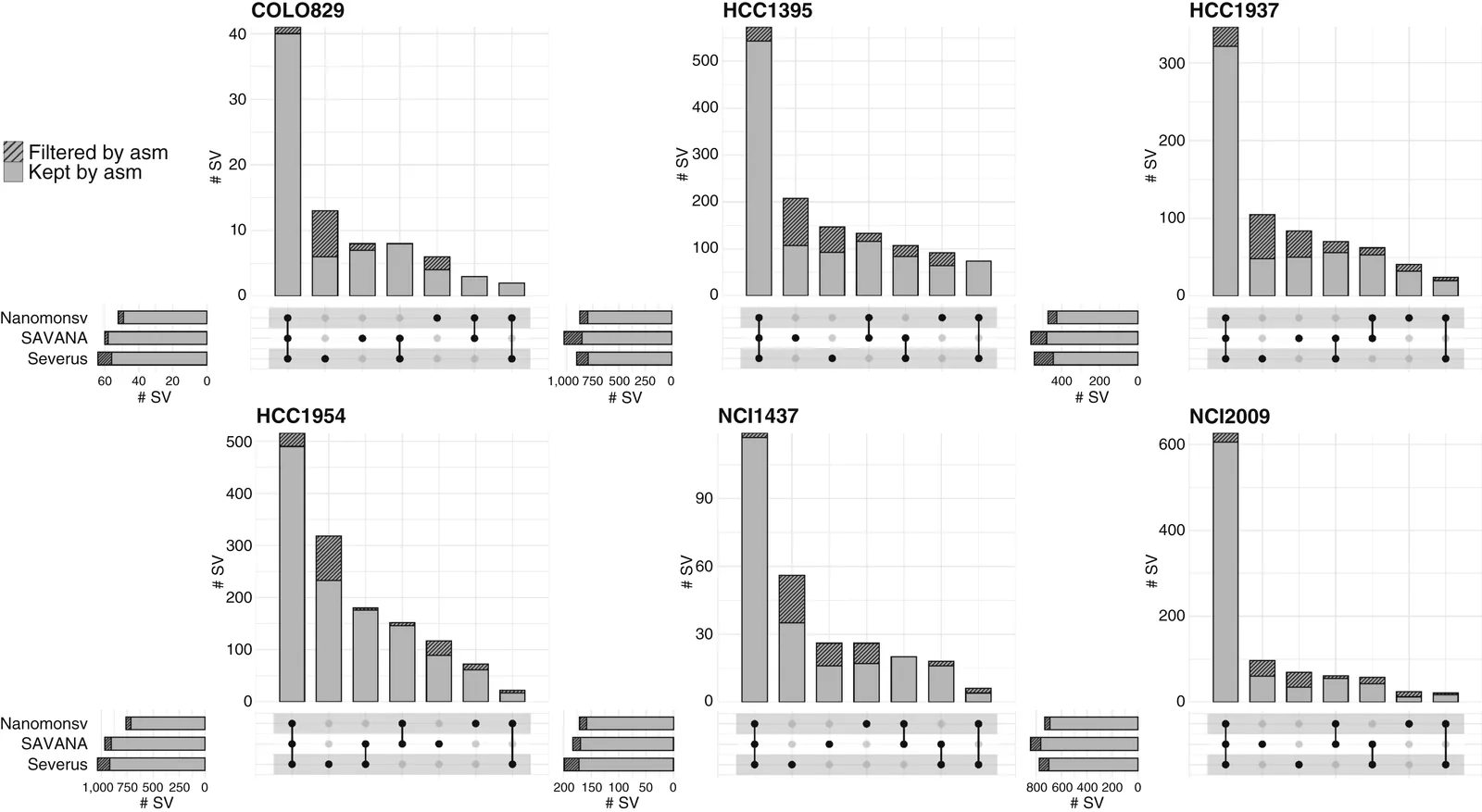
Intersection of somatic SVs called by three callers from six tumor–normal pairs before and after filtering by self-assembly (asm).

We ran all SV callers filtering in three modes: “l,” “l + s,” and “l + g,” where “l” denotes raw SV calls from the linear reference genome only, “l + s” filters raw calls with self-assembly, and “l + g” filters raw calls with pangenome. The self-assembly filter “l + s” reduces the number of SV calls (Fig. 4), especially for SV calls specific to one caller (Fig. 5). Consistent with the evaluation on COLO829 data, the pangenome filter “l + g” reduces the number of SV calls further (Supplementary Figs. S4 and S5). Most of the calls only filtered by “l + g” fall in tandem repeats, which often overlap with germline SVs in the pangenome and thus get filtered.

Severus, SAVANA, and nanomonsv generally agree with each other for most somatic SVs identified by all three callers after the filtering (Fig. 5; Supplementary Fig. S6). The great majority of SVs called by all three tools are also supported by self-assemblies and are likely to be accurate. On the contrary, SVs called by one or two callers are less reliable.

### Assessing somatic SV calling for patients with osteosarcoma

Benchmarks above focused on cancer cell lines, but primary tumors from patient samples tend to be more heterogeneous. To assess the impact of tumor purity on the somatic SV filtering, we analyzed five tumor–normal pairs from an osteosarcoma long-read cohort obtained from European Genome-phenome Archive (EGA; Table 1; ref. 13). We could not assemble the normal samples well (Supplementary Table S2) due to the high sequencing error rates, as they were sequenced with the older SQK-LSK110 kit. We thus only applied “l” (GRCh38 only) and “l + g” (GRCh38 with pangenome graph filtering) for SV calling.

**Figure 6.**
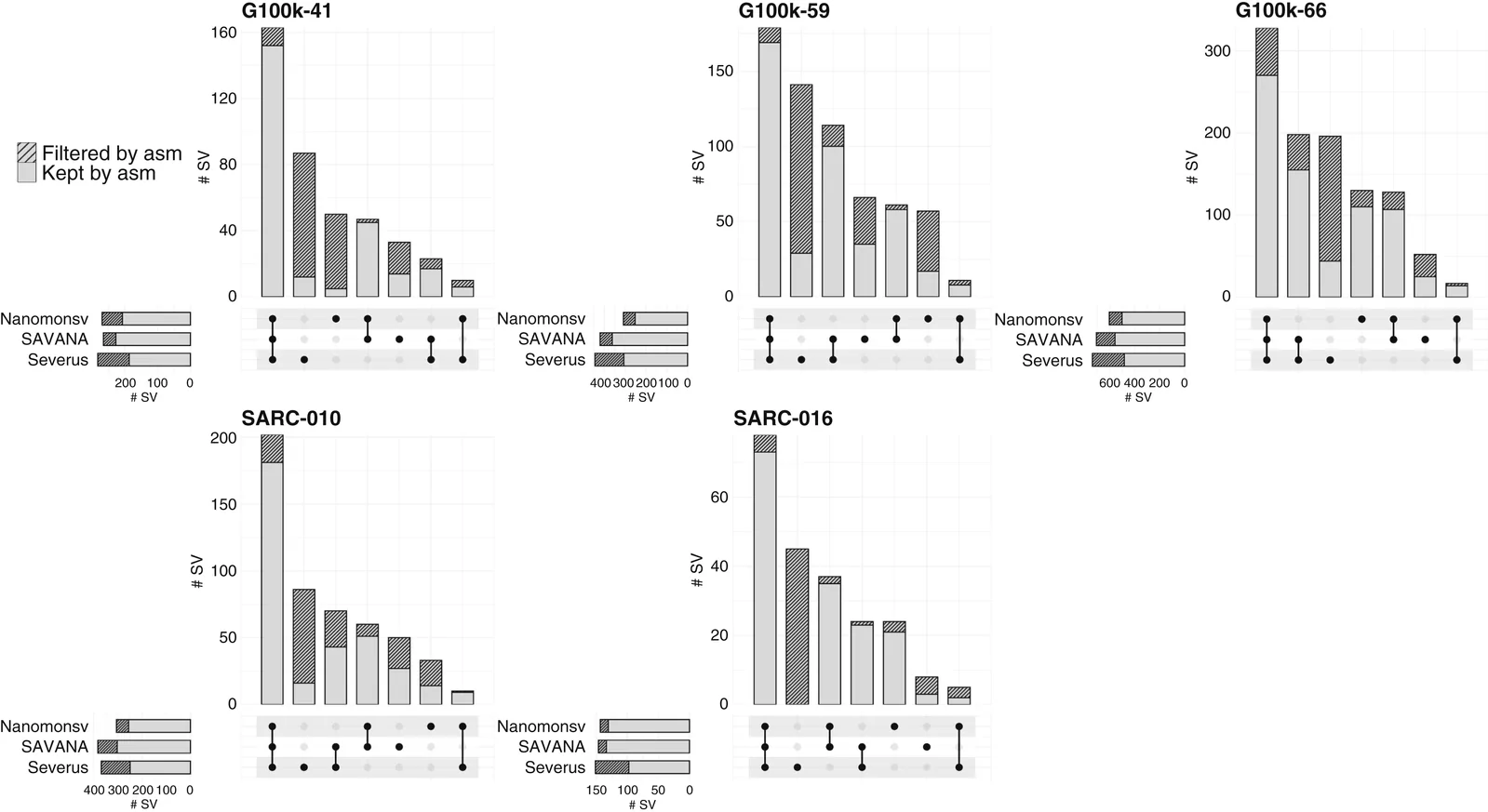
Intersection of somatic SVs called by three callers from five tumor–normal pairs in the osteosarcoma long-read cohort before and after filtering by pangenome alignment (graph). asm, assembly.

Consistent with the cell line–based results, the consensus call sets across all three callers showed the fewest filtered SVs, whereas among caller-specific call sets, Severus consistently had the largest number of filtered SVs in its unique calls, followed by SAVANA and nanomonsv (Fig. 6; Supplementary Fig. S3).

## Discussion

Minisv is unique in its use of alignment against multiple genomes, including pangenome and the self-assembly of normal samples. It greatly reduces alignment artifacts caused by germline SVs relative to the reference genome and thus improves the specificity of somatic or mosaic SV calls with little loss in sensitivity.

Applying our method to multiple datasets, we found that SV callers specialized for tumor–normal pairs, which include nanomonsv, Severus, and SAVANA, all work reasonably well in that most calls are shared by all callers. Although Sniffles2 supports somatic SV calling from tumor–normal pairs, it does not compete with dedicated tumor SV callers. Self-assembly–based filtering improves the accuracy, and this improvement is robust to sequencing depth in our downsampling analyses. Calling mosaic SVs of low allele fraction is much more challenging. All current methods would report many germline or artifactual SVs as mosaic. The self-assembly filter is particularly powerful in this setting and is highly recommended. The effectiveness of assembly-based filtering depends on the quality of the assembly. When the assembly quality is low, pangenome-based filtering can be used although this approach may filter out true somatic SVs that overlap with germline SVs in populations. The representativeness of the reference panel will also affect the power of pangenome-based filtering.

A potential concern with the self-assembly filter is that somatic SVs of high allele fraction may be represented in the assembly and may be mistakenly filtered with our method. A better solution is to keep a candidate somatic SV call if it only occurs in one haplotype. Taking advantage of haplotype phasing will also improve mosaic SV calling in normal tissues. Another weakness of our current implementation is that we need to align all reads against multiple genomes or pangenomes. This will increase the total runtime but can be alleviated by only aligning reads containing candidate SV calls. We are developing an integrated long-read variant caller that will address these technical issues and supposedly achieve higher accuracy and performance.

## Supplementary Material

Supplementary Figure 1 Somatic SV calling on matched COLO829 tumor-normal cell lines after downsampling the reads to 50% of the original sequences. A–D. metrics on de novo assembly-based filtering, A. Number of false positive (FP) and true positive (TP) SV calls (C) from PacBio HiFi data at different cutoffs on supporting reads. The solid bars correspond to SV calls still observed in the alignment against the COLO829BL (derived from blood) assembly, while the shaded bars correspond to calls filtered by the assembly. The horizontal line gives the number of ground truth SV calls. B. Number of FP and TP calls (D) from ONT reads.

Supplementary Figure 2 gnomAD filtering analysis of the somatic SVs in COLO829 HiFi sequencing data. The SV calls were filtered at supported read cutoff of 3. The left column show the performance from HiFi sequencing data, right column shows the nanopore dataset performance.

Supplementary Figure 3 Effect of assembly-based filtering on SV calling on six tumor-normal pairs after downsampling the sequence to 50% of the original depth. The number on the top indicates the number of supporting reads required to call an SV.

Supplementary Figure 4 Intersection of somatic SVs called by three callers from six tumor-normal pairs before and after filtering by HPRC v2 pangenome.

Supplementary Figure 5 Intersection of somatic SVs called by three callers from six tumor-normal pairs before and after filtering by HPRC2 pangenome based on the downsampled (50%) datasets.

Supplementary Figure 6 Intersection of somatic SVs called by three callers from six tumor-normal pairs before and after filtering by self-assembly based on the downsampled (50%) datasets.

Supplementary Table 1 Summary of software source codes and reference genomes.

Supplementary Table 2 Assembly quality evaluation metrics of all samples in this study.

Supplementary Table 3 Raw SV calls stratified by size

Supplementary Table 4 Assembly-filtered SV calls stratified by size

## Data Availability

The genome references used in the evaluation are listed in the summary table (Supplementary Table S1). Both HCC1395 (RRID: CVCL_1249) and COLO829 (RRID: CVCL_1137) human cancer cell lines, along with their paired normal cell lines, HiFi sequencing reads were obtained from the PacBio Cloud website. The rest of the human cancer cell lines and paired lymphoblast normal cell lines’ PacBio HiFi and ONT R10 sequencing data were obtained from the NCBI Sequence Read Archive (SRA) database [SRP494767 (10)]. The paired normal sample PacBio HiFi sequencing data from the human pangenome reference project are available from the NCBI SRA database [SRP305758 (16)] and Amazon Cloud. All the involved data are listed in the summary table (Table 1); we downloaded these SRA datasets using fastq-dump. All the SV calls and *de novo* assemblies are deposited in Zenodo (https://doi.org/10.5281/zenodo.14715664) at https://zenodo.org/uploads/21852833. We also randomly collected primary patient tumor–normal paired long-read ONT WGS data from the osteosarcoma cohort deposited in EGA [accession ID: EGAD50000000925 (13)] using the EGA outbox SFTP site (Table 1).

## Code Availability

The JavaScript version of minisv is available at https://github.com/lh3/minisv, with additional functionality implemented in https://github.com/qinqian/minisv.py. The other SV callers, aligners, converters, and assembler software, along with the dependent data of the VNTR BED file and minisv *eval* genome region BED file included in this study, are listed in Supplementary Table S1. The source codes to process, evaluate, and benchmark different SV callers are available at https://github.com/qinqian/pangenome_sv_benchmarking.

## References

[1] Quinlan AR , Hall IM. Characterizing complex structural variation in germline and somatic genomes. Trends Genet 2012;28:43–53. 10.1016/j.tig.2011.10.002. PMC3249479 22094265

[2] Cosenza MR , Rodriguez-Martin B, Korbel JO. Structural variation in cancer: role, prevalence, and mechanisms. Annu Rev Genomics Hum Genet 2022;23:123–52. 10.1146/annurev-genom-120121-101149. 35655332

[3] Hanahan D . Hallmarks of cancer-then and now, and beyond. Cell 2026;189:2254–77. 10.1016/j.cell.2025.12.049. 41616779

[4] Hanahan D , Weinberg RA. Hallmarks of cancer: the next generation. Cell 2011;144:646–74. 10.1016/j.cell.2011.02.013. 21376230

[5] Li Y , Roberts ND, Wala JA, Shapira O, Schumacher SE, Kumar K, . Patterns of somatic structural variation in human cancer genomes. Nature 2020;578:112–21. 10.1038/s41586-019-1913-9. PMC7025897 32025012

[6] ICGC/TCGA Pan-Cancer Analysis of Whole Genomes Consortium . Pan-cancer analysis of whole genomes. Nature 2020;578:82–93. 10.1038/s41586-020-1969-6. PMC7025898 32025007

[7] Benjamini Y , Speed TP. Summarizing and correcting the GC content bias in high-throughput sequencing. Nucleic Acids Res 2012;40:e72. 10.1093/nar/gks001. PMC3378858 22323520

[8] Dohm JC , Lottaz C, Borodina T, Himmelbauer H. Substantial biases in ultra-short read data sets from high-throughput DNA sequencing. Nucleic Acids Res 2008;36:e105. 10.1093/nar/gkn425. PMC2532726 18660515

[9] Wenger AM , Peluso P, Rowell WJ, Chang P-C, Hall RJ, Concepcion GT, . Accurate circular consensus long-read sequencing improves variant detection and assembly of a human genome. Nat Biotechnol 2019;37:1155–62. 10.1038/s41587-019-0217-9. PMC6776680 31406327

[10] Marx V . Method of the year: long-read sequencing. Nat Methods 2023;20:6–11. 10.1038/s41592-022-01730-w. 36635542

[11] Chaisson MJP , Huddleston J, Dennis MY, Sudmant PH, Malig M, Hormozdiari F, . Resolving the complexity of the human genome using single-molecule sequencing. Nature 2015;517:608–11. 10.1038/nature13907. PMC4317254 25383537

[12] Keskus AG , Bryant A, Ahmad T, Yoo B, Aganezov S, Goretsky A, . Severus detects somatic structural variation and complex rearrangements in cancer genomes using long-read sequencing. Nat Biotechnol 2026;44:247–57. 10.1038/s41587-025-02618-8. PMC12483193 40185952

[13] Elrick H , Sauer CM, Espejo Valle-Inclan J, Trevers K, Tanguy M, Zumalave S, . SAVANA: reliable analysis of somatic structural variants and copy number aberrations using long-read sequencing. Nat Methods 2025;22:1436–46. 10.1038/s41592-025-02708-0. PMC12240814 40437218

[14] Lin J , Wang S, Audano PA, Meng D, Flores JI, Kosters W, . SVision: a deep learning approach to resolve complex structural variants. Nat Methods 2022;19:1230–3. 10.1038/s41592-022-01609-w. PMC9985066 36109679

[15] Smolka M , Paulin LF, Grochowski CM, Horner DW, Mahmoud M, Behera S, . Detection of mosaic and population-level structural variants with Sniffles2. Nat Biotechnol 2024;42:1571–80. 10.1038/s41587-023-02024-y. PMC11217151 38168980

[16] Shiraishi Y , Koya J, Chiba K, Okada A, Arai Y, Saito Y, . Precise characterization of somatic complex structural variations from tumor/control paired long-read sequencing data with nanomonsv. Nucleic Acids Res 2023;51:e74. 10.1093/nar/gkad526. PMC10415145 37336583

[17] Liao W-W , Asri M, Ebler J, Doerr D, Haukness M, Hickey G, . A draft human pangenome reference. Nature 2023;617:312–24. 10.1038/s41586-023-05896-x. PMC10172123 37165242

[18] Cheng H , Concepcion GT, Feng X, Zhang H, Li H. Haplotype-resolved de novo assembly using phased assembly graphs with hifiasm. Nat Methods 2021;18:170–5. 10.1038/s41592-020-01056-5. PMC7961889 33526886

[19] Cheng H , Qu H, McKenzie S, Lawrence KR, Windsor R, Vella M, . Efficient near-telomere-to-telomere assembly of nanopore simplex reads. Nature 2026;655:166–73. 10.1038/s41586-026-10105-6. PMC13070018 41639459

[20] Li H . Minimap2: pairwise alignment for nucleotide sequences. Bioinformatics 2018;34:3094–100. 10.1093/bioinformatics/bty191. PMC6137996 29750242

[21] Li H , Feng X, Chu C. The design and construction of reference pangenome graphs with minigraph. Genome Biol 2020;21:265. 10.1186/s13059-020-02168-z. PMC7568353 33066802

[22] Espejo Valle-Inclan J , Besselink NJM, De Bruijn E, Cameron DL, Ebler J, Kutzera J, . A multi-platform reference for somatic structural variation detection. Cell Genomics 2022;2:100139. 10.1016/j.xgen.2022.100139. PMC9903816 36778136

[23] Velazquez-Villarreal EI , Maheshwari S, Sorenson J, Fiddes IT, Kumar V, Yin Y, . Single-cell sequencing of genomic DNA resolves sub-clonal heterogeneity in a melanoma cell line. Commun Biol 2020;3:318. 10.1038/s42003-020-1044-8. PMC7316972 32587328

[24] Qin Q , Li H. Challenges in structural variant calling in low-complexity regions. GigaScience 2025;14:giaf154. 10.1093/gigascience/giaf154. PMC12758381 41384802

