# Supplementary material for: Improving Long-Read Somatic Structural Variant Calling with Pangenome and De Novo Personal Genome Assembly
Source: Cancer Res Commun. 2026 Sep 29;6(9):2282–90. doi: 10.1158/2767-9764.CRC-25-0769 (PMC13621377; doi:10.1158/2767-9764.CRC-25-0769)
Supplement: Supplementary file 1 [file crc-25-0769_supplementary_figure_1_suppsf1.pdf]

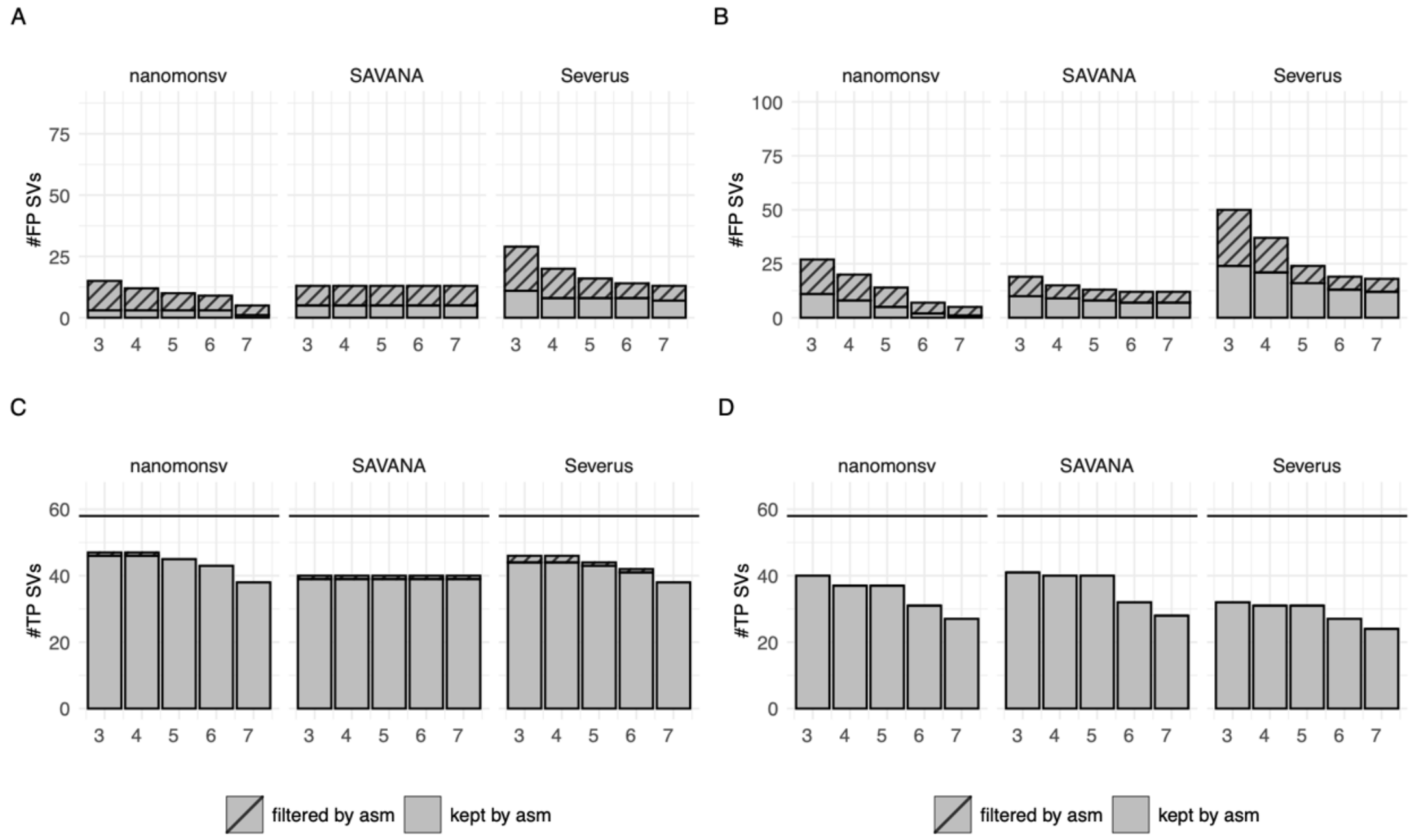

Supplementary Figure 1. Somatic SV calling on matched COLO829 tumor-normal cell lines after downsampling the reads to 50% of the original sequences. A–D. metrics on de novo assembly-based filtering, **A.** Number of false positive (FP) and true positive (TP) SV calls **(C)** from PacBio HiFi data at different cutoffs on supporting reads. The solid bars correspond to SV calls still observed in the alignment against the COLO829BL (derived from blood) assembly, while the shaded bars correspond to calls filtered by the assembly. The horizontal line gives the number of ground truth SV calls. **B.** Number of FP and TP calls **(D)** from ONT reads.
