# Supplementary material for: Improving Long-Read Somatic Structural Variant Calling with Pangenome and De Novo Personal Genome Assembly
Source: Cancer Res Commun. 2026 Sep 29;6(9):2282–90. doi: 10.1158/2767-9764.CRC-25-0769 (PMC13621377; doi:10.1158/2767-9764.CRC-25-0769)
Supplement: Supplementary file 2 [file crc-25-0769_supplementary_figure_2_suppsf2.pdf]

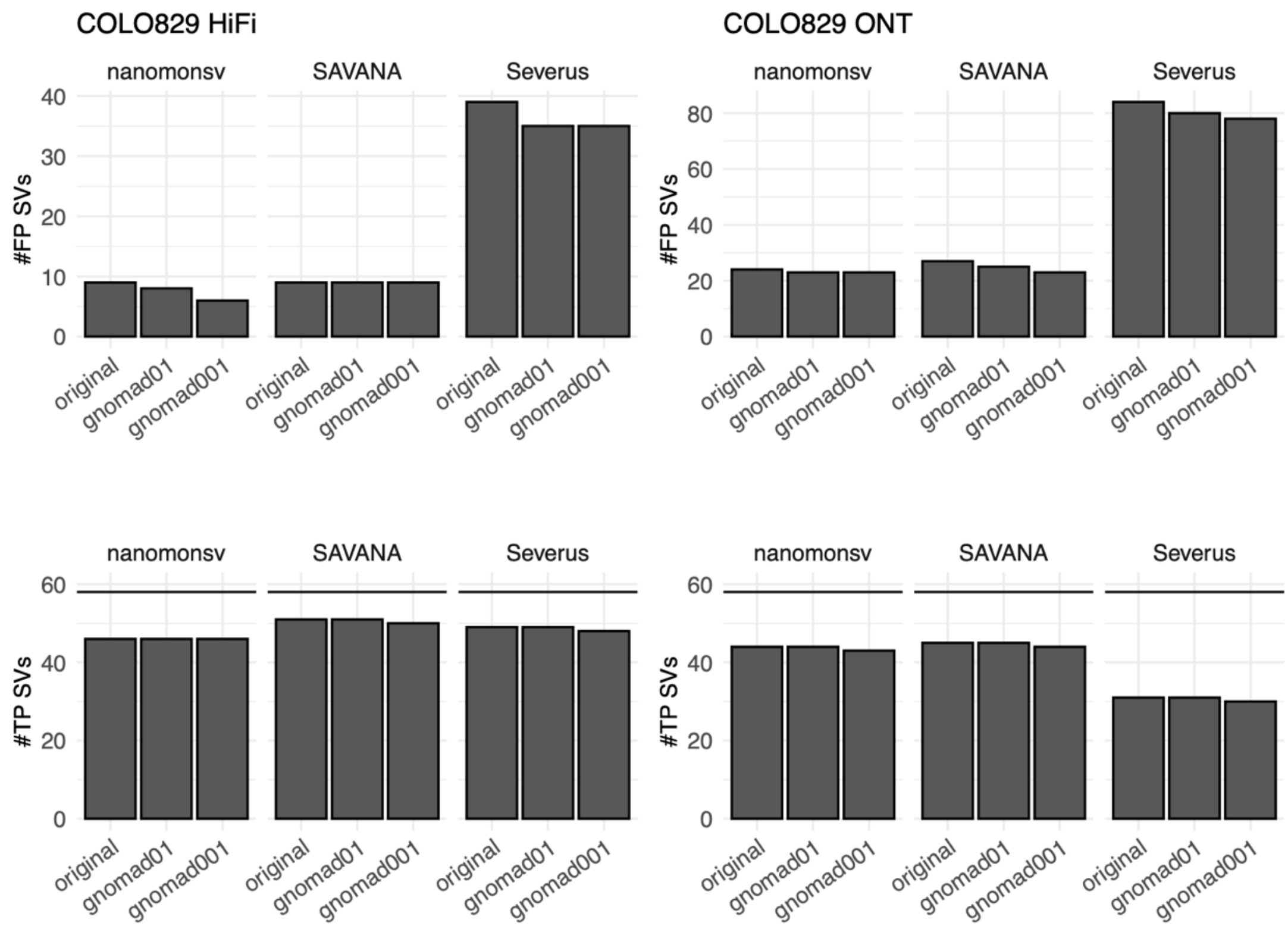

Supplementary Figure 2. gnomAD filtering analysis of the somatic SVs in COLO829 HiFi sequencing data. The SV calls were filtered at supported read cutoff of 3. The left column show the performance from HiFi sequencing data, right column shows the nanopore dataset performance.
