# Supplementary material for: Improving Long-Read Somatic Structural Variant Calling with Pangenome and De Novo Personal Genome Assembly
Source: Cancer Res Commun. 2026 Sep 29;6(9):2282–90. doi: 10.1158/2767-9764.CRC-25-0769 (PMC13621377; doi:10.1158/2767-9764.CRC-25-0769)
Supplement: Supplementary file 3 [file crc-25-0769_supplementary_figure_3_suppsf3.pdf]

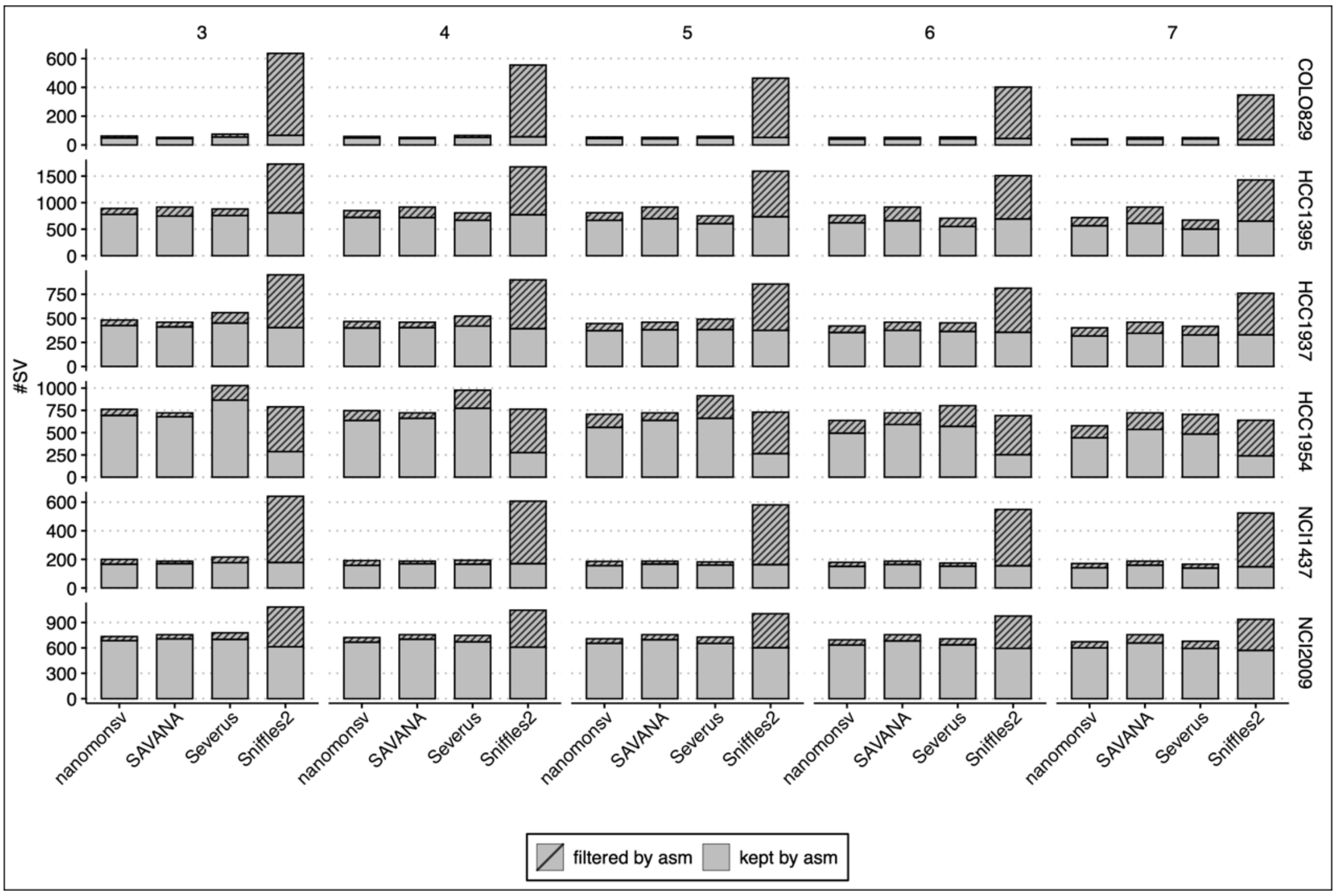

Supplementary Figure 3. Effect of assembly-based filtering on SV calling on six tumor-normal pairs after downsampling the sequence to 50% of the original depth. The number on the top indicates the number of supporting reads required to call an SV.
