# Supplementary material for: Improving Long-Read Somatic Structural Variant Calling with Pangenome and De Novo Personal Genome Assembly
Source: Cancer Res Commun. 2026 Sep 29;6(9):2282–90. doi: 10.1158/2767-9764.CRC-25-0769 (PMC13621377; doi:10.1158/2767-9764.CRC-25-0769)
Supplement: Supplementary file 5 [file crc-25-0769_supplementary_figure_5_suppsf5.pdf]

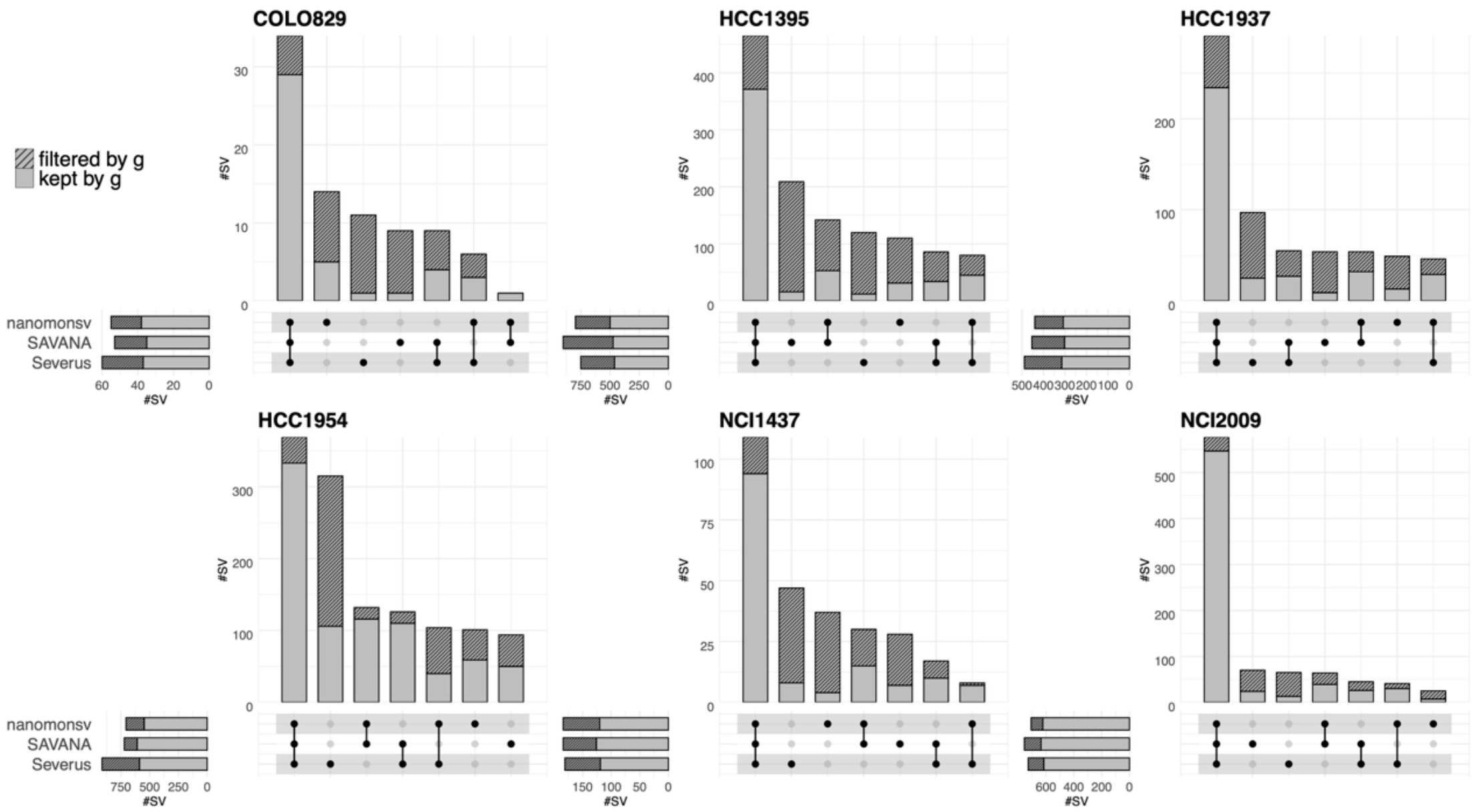

Supplementary Figure 5. Intersection of somatic SVs called by three callers from six tumor-normal pairs before and after filtering by HPRC2 pangenome based on the downsampled (50%) datasets.
