# Supplementary material for: Improving Long-Read Somatic Structural Variant Calling with Pangenome and De Novo Personal Genome Assembly
Source: Cancer Res Commun. 2026 Sep 29;6(9):2282–90. doi: 10.1158/2767-9764.CRC-25-0769 (PMC13621377; doi:10.1158/2767-9764.CRC-25-0769)
Supplement: Supplementary file 7 [file crc-25-0769_supplementary_table_1_suppst1.pdf]

Supplementary Table 1. Summary of software source codes and reference genomes.

Code and Data	Source link
**Severus**	https://github.com/KolmogorovLab/Severus
**NanomonSV**	https://github.com/friend1ws/nanomonsv
**SAVANA**	https://github.com/cortes-ciriano-lab/savana
**Sniffles2**	https://github.com/fritzsedlazeck/Sniffles
**Hifiasm**	https://github.com/chhylp123/hifiasm
**Samtools**	https://github.com/samtools/samtools
**Minimap2**	https://github.com/lh3/minimap2
**Minigraph**	https://github.com/lh3/minigraph
**GRCh38**	ftp://ftp.ncbi.nlm.nih.gov/genomes/all/GCA/000/001/405/GCA_000001405.15_GRCh38/seqs_for_alignment_pipelines.ucsc_ids/GCA_000001405.15_GRCh38_no_alt_analysis_set.fna.gz
**Chm13v2**	https://s3-us-west-2.amazonaws.com/human-pangenomics/T2T/CHM13/assemblies/analysis_set/chm13v2.0.fa.gz
**CHM13 graph**	https://zenodo.org/records/16728828/files/CHM13-464.gfa.gz
