# Supplementary material for: Improving Long-Read Somatic Structural Variant Calling with Pangenome and De Novo Personal Genome Assembly
Source: Cancer Res Commun. 2026 Sep 29;6(9):2282–90. doi: 10.1158/2767-9764.CRC-25-0769 (PMC13621377; doi:10.1158/2767-9764.CRC-25-0769)
Supplement: Supplementary file 8 [file crc-25-0769_supplementary_table_2_suppst2.pdf]

Supplementary Table 2. Assembly quality evaluation metrics of all samples in this study.

Sample	hap1_sz	hap1_n50	hap2_sz	hap2_n50
**COLO829BL**	3019286100	71440721	2962318057	53115481
**COLO829BL_ont1**	2900465495	110392276	3079232008	100883984
**HCC1395BL**	2974967368	24369538	2838849942	18010134
**HCC1937BL**	2959991875	85197473	2984350385	81625214
**HCC1954BL**	3033056935	63107572	3015800995	81348949
**NCI1437BL**	3047746475	89289166	2977726587	77600242
**NCI2009BL**	3015854156	70830962	3034798552	74546332
**downCOLO829BL.hifi1**	2989072955	31464604	2981555556	42399477
**downCOLO829BL.ont1**	3049024541	29955129	2926530162	28330913
**downHCC1395BL.hifi1**	2977189635	9326668	2792576140	6425923
**downHCC1937BL.hifi1**	2970824406	41418257	2968402598	50486419
**downHCC1954BL.hifi1**	3020709233	59784151	3011336701	41123151
**downNCI1437BL.hifi1**	3027550349	62343250	2952048054	52494192
**downNCI2009BL.hifi1**	2998607926	51238250	3036048858	53419297
**G100k-41_normal**	1597442570	173883	457570851	299023
**G100k-59_normal**	2193287005	544953	1105945627	704736
**G100k-66_normal**	1974387402	2025543	1808788895	2457458
**SARC-010_normal**	1978414399	1822396	1801650663	2394620
**SARC-016_normal**	2036668627	2314996	1678314447	2717733
