# Supplementary material for: Improving Long-Read Somatic Structural Variant Calling with Pangenome and De Novo Personal Genome Assembly
Source: Cancer Res Commun. 2026 Sep 29;6(9):2282–90. doi: 10.1158/2767-9764.CRC-25-0769 (PMC13621377; doi:10.1158/2767-9764.CRC-25-0769)
Supplement: Supplementary file 9 [file crc-25-0769_supplementary_table_3_suppst3.pdf]

Supplementary Table 3. Raw SV calls stratified by size

Tool	SizeBin	FN tot	FN	Recall	FP tot	FP	Precision	F1
**nanomonsv**	50-100	1	1	0	0	0	1	0
	100-500	2	0	1	5	3	0.4	0.571
	500-1000	2	1	0.5	1	0	1	0.667
	1k-10k	10	2	0.8	12	4	0.667	0.727
	10k-20k	3	0	1	4	1	0.75	0.857
	20k-100k	11	3	0.727	8	0	1	0.842
	>100k	29	5	0.828	25	1	0.96	0.889
**SAVANA**	50-100	1	1	0	0	0	1	0
	100-500	2	0	1	2	0	1	1
	500-1000	2	1	0.5	2	1	0.5	0.5
	1k-10k	10	1	0.9	11	2	0.818	0.857
	10k-20k	3	0	1	3	0	1	1
	20k-100k	11	1	0.909	11	1	0.909	0.909
	>100k	29	3	0.897	31	5	0.839	0.867
**Severus**	50-100	1	1	0	1	1	0	NA
	100-500	2	0	1	24	22	0.083	0.154
	500-1000	2	1	0.5	6	5	0.167	0.250
	1k-10k	10	0	1	32	22	0.312	0.476
	10k-20k	3	0	1	9	6	0.333	0.500
	20k-100k	11	1	0.9091	14	4	0.714	0.800
	>100k	29	6	0.7931	42	19	0.548	0.648
