# Supplementary material for: Improving Long-Read Somatic Structural Variant Calling with Pangenome and De Novo Personal Genome Assembly
Source: Cancer Res Commun. 2026 Sep 29;6(9):2282–90. doi: 10.1158/2767-9764.CRC-25-0769 (PMC13621377; doi:10.1158/2767-9764.CRC-25-0769)
Supplement: Supplementary file 10 [file crc-25-0769_supplementary_table_4_suppst4.pdf]

Supplementary Table 4. Assembly-filtered SV calls stratified by size

Tool	SizeBin	FN tot	FN	Recall	FP tot	FP	Precision	F1
**nanomonsv**	50-100	1	1	0	0	0	1	0
	100-500	2	0	1	3	1	0.6667	0.800
	500-1000	2	1	0.5	1	0	1	0.667
	1k-10k	10	2	0.8	11	3	0.7273	0.762
	10k-20k	3	0	1	4	1	0.75	0.857
	20k-100k	11	3	0.727	8	0	1	0.842
	>100k	29	5	0.828	24	0	1	0.906
**SAVANA**	50-100	1	1	0	0	0	1	0
	100-500	2	0	1	2	0	1	1
	500-1000	2	1	0.5	2	1	0.5	0.5
	1k-10k	10	1	0.9	10	1	0.9	0.9
	10k-20k	3	0	1	3	0	1	1
	20k-100k	11	1	0.909	11	1	0.909	0.909
	>100k	29	3	0.897	30	4	0.867	0.881
**Severus**	50-100	1	1	0	0	0	1	0
	100-500	2	0	1	5	3	0.4	0.571
	500-1000	2	1	0.5	2	1	0.5	0.5
	1k-10k	10	0	1	15	5	0.667	0.800
	10k-20k	3	0	1	5	2	0.6	0.75
	20k-100k	11	1	0.909	13	3	0.769	0.833
	>100k	29	6	0.793	27	4	0.852	0.821
